# Clinical applicability of cardiac magnetic resonance feature tracking analysis in patients with atrial fibrillation undergoing ablation

**DOI:** 10.1186/1532-429X-16-S1-P154

**Published:** 2014-01-16

**Authors:** Felix Ceelen, Ross J Hunter, Redha Boubertakh, Wieland H Sommer, Richard Schilling, Steffen E Petersen

**Affiliations:** 1 Centre for Advanced Cardiovascular Imaging, Queen Mary, University of London, London, UK; 2 Department of Clinical Radiology, University of Munich, Munich, Germany

## Background

Catheter-based ablation techniques are widely applied in the restoration of sinus rhythm. The latter may result in an improvement of left heart function in patients with atrial fibrillation (AF). Cardiovascular magnetic resonance (CMR) feature tracking (FT) might help detect subtle wall-motion abnormalities. We aimed to analyze existence and reversibility of subtle cardiac dysfunction following atrial fibrillation ablation.

## Methods

**Figure 1:**
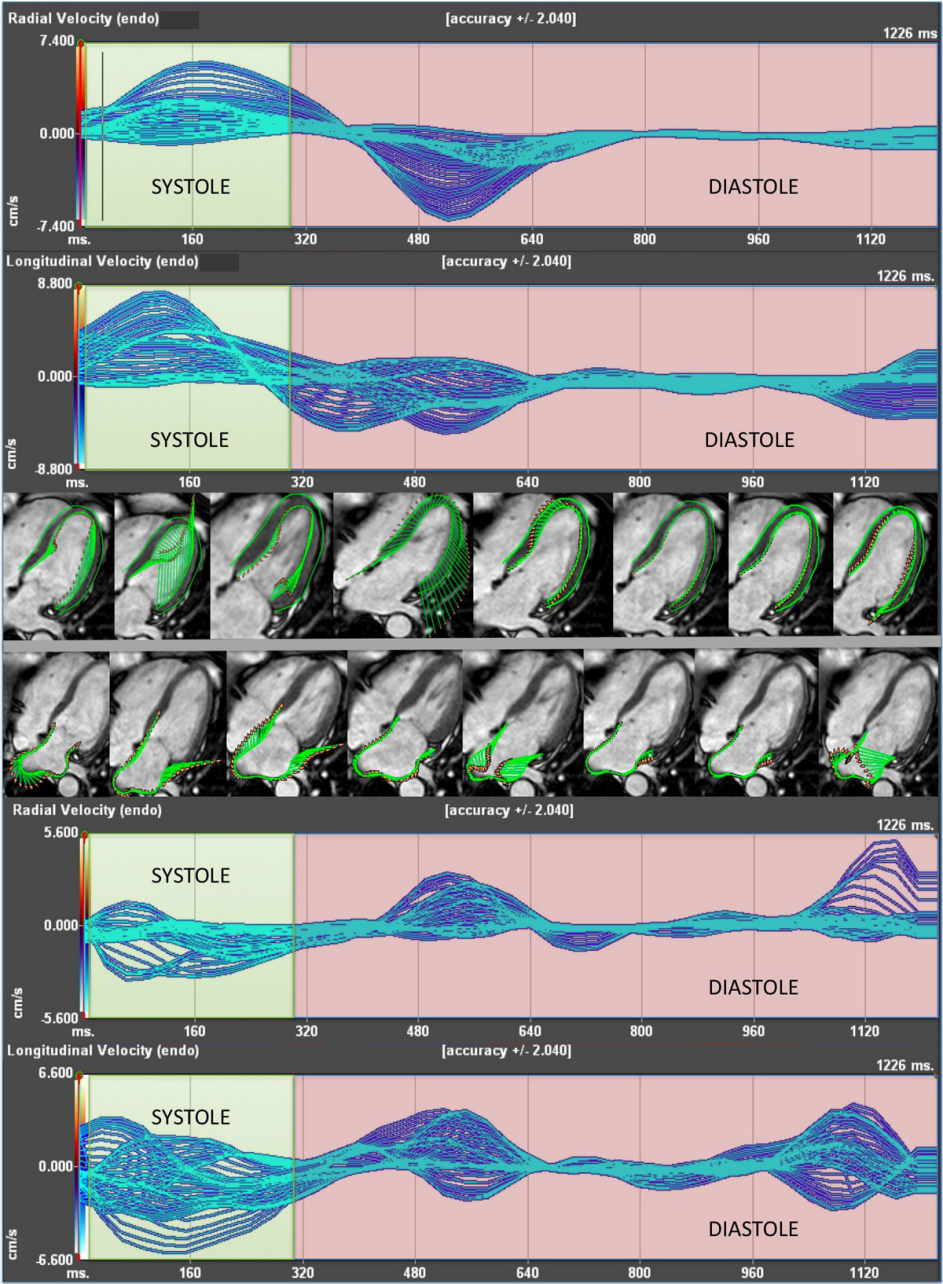


We studied 28 consecutive patients (mean age: 61 years) with paroxysmal AF who underwent pulmonary vein isolation (PVI). CMR imaging was done 3 (± 3) days before and 3.4 (± 1.1) months after ablation. FT analysis was performed in order to assess left heart function. Three-dimensional peak velocities, strain, strain rate and torsion were measured before ablation and compared to post ablation. Systolic peaks were separated from diastolic reverse peaks in order to better evaluate left heart function (See Figure 1). A meta-analysis was performed to compare published healthy volunteers' tracking data to our patients before AF ablation. Statistical analysis included paired student's t-test and random effects metaanalysis and Bland-Altman analysis.

## Results

17 patients (61%) were free from AF at follow-up. Bland-Altman analysis showed good coefficients of variation. Of all 195 parameters, 27 changed (14%): 9 improved significantly (5%), 12 worsened significantly (6%), whereas 6 parameters worsened not significantly (3%). 18 of 120 systolic parameters changed (15%), 14 worsened (12%), 4 improved (3%). In 9 of 75 diastolic parameters, values changed (12%): 5 improved (7%) and 4 worsened (5%). Meta-analysis revealed that our collective's FT values at baseline did not differ significantly from healthy volunteers' values (Q values of 0.01 [p value 0.921] and 1.499 [p value 0.221]).

## Conclusions

AF patients undergoing ablation appear to have near normal cardiac wall motion, which does not improve following successful ablation. Feature tracking analysis is a reliable tool to determine treatment effects but is more likely to show positive findings if the population is unhealthy.

## Funding

This work forms part of the research themes contributing to the translational research portfolio of Barts Cardiovascular Biomedical Research Unit which is supported and funded by the National Institute for Health Research.

